# Inhibition of non-small cell lung cancer metastasis by knocking down APE1 through regulating myeloid-derived suppressor cells-induced immune disorders

**DOI:** 10.18632/aging.205938

**Published:** 2024-06-14

**Authors:** Zhenlong Zhang, Yuchen Lin, Xiaojie Pan, Shuchen Chen

**Affiliations:** 1 The Affiliated Hospital, Fujian Medical University, Fuzhou 362002, China; 2 The Shengli Clinical Medical College, Fujian Medical University, Fujian Provincial Hospital, Fuzhou 350001, China

**Keywords:** NSCLC, MDSCs, APE1, A549, tumor immune

## Abstract

Background: Non-small cell lung cancer (NSCLC) represents a highly immunogenic malignancy. Immunologic tolerance facilitated by myeloid-derived suppressor cells (MDSCs) is implicated in primary or secondary resistance mechanisms in NSCLC. The potential role of APE1 in regulating NSCLC metastasis by targeting MDSCs remains uncertain.

Methods: This study utilized a plasmid, Plxpsp-mGM-CSF, to induce elevated granulocyte-macrophage colony-stimulating factor (GM-CSF) expression in A549 cells. Tumor transplantation experiments involved A549, A549+GM-CSF, and A549+GM-CSF-siAPE1 cell lines. Evaluation encompassed MDSCs, Treg cells, IgG, CD3, and CD8 levels.

Results: Notably, lung cancer tissues and cells displayed markedly reduced APE1 expression. siAPE1 transfection significantly curtailed tumor growth compared to the A549+GM-CSF group. APE1 knockdown orchestrated immune system modulation in lung tumor mice, characterized by diminished MDSCs but augmented Treg cells, IgG, CD3, and CD8. Additionally, APE1 knockdown led to reduced levels of pro-MDSC cytokines (HGF, CCL5, IL-6, CCL12) and a concurrent upregulation of the anti-MDSC cytokine IL-1ra. Furthermore, APE1 knockdown impeded cell viability in both A549 and H1650 cells.

Conclusions: Transplantation of A549-GM-CSF amplified MDSC levels, fostering accelerated tumor growth, while mitigating MDSC levels through APE1 knockdown hindered tumor progression and alleviated inflammatory infiltration in lung cancer tissues. Strategies targeting the APE1/MDSC axis offer a promising approach for lung cancer prevention and treatment, presenting novel insights for NSCLC management.

## INTRODUCTION

Lung cancer has the highest incidence and mortality rates in China, ranking as the leading and second-leading cancer among both men and women, respectively [1]. Clinically, non-small cell lung cancer (NSCLC) and small cell lung cancer (SCLC) are divided into two categories based on their pathological and histological characteristics. NSCLC accounts for about 80%, and due to the diversity of pathological subtypes, heterogeneity between subtypes, and drug resistance during treatment, the therapeutic efficacy of this major category of diseases is poor [2]. The advent of immune checkpoint inhibitors (ICIs) has significantly advanced the drug treatment for advanced-stage lung cancer [3]. One of the characteristics of the tumor microenvironment and tumor cells is immune suppression and immune escape [4].

Myeloid-derived suppressor cells (MDSCs) originate from bone marrow progenitor cells and immature myeloid cells [5]. Under normal physiological conditions, these cells serve as precursors to granulocytes, dendritic cells, and macrophages, rapidly differentiating into mature forms and entering the corresponding tissues and organs to perform normal immune functions [6]. However, in the context of malignant tumors, the maturation of these myeloid lineage-derived precursor cells is hindered, leading to their stagnation at various stages of differentiation [3]. Consequently, they accumulate in the bone marrow, spleen, peripheral blood, lymph nodes, and tumor lesions. Regulation of MDSCs has been viewed as a potential therapeutic strategy for tumor treatment [7]. In various malignant tumors, such as colorectal cancer, breast cancer, bladder cancer, melanoma, and non-small cell lung cancer, the quantity of MDSCs is positively correlated with the clinical staging of patients and tumor burden, while it is negatively associated with patient prognosis [8, 9]. MDSCs also exhibit a negative correlation with the effectiveness of immunotherapy.

APE1 plays a crucial role in cellular vitality and embryonic development, and it also possesses DNA repair activity [10, 11]. Indeed, APE1 functions as a redox effector for many transcription factors (such as NF-κB, HIF-1α, STAT-3, PAX8, AP-1, and p53), playing a key role in mediating critical genes involved in tumor progression or drug resistance [12, 13]. Furthermore, the abnormal overexpression of APE1 promotes the secretion of TGF-β, initiating epithelial-mesenchymal transition in tumor cells and reducing the cytotoxic response of cells to drugs, resulting in poor prognosis for cancer patients [14]. However, if APE1 could affect the NSCLC metastasis through targeting MDSCs remains unclear.

We searched for the expression of APE1 in LUAD and LUSC through GEPIA and TCGA databases, and observed the relationship between APE1 expression and tumor staging and survival rate. Further validate the high expression of APE1 in tumor tissue by detecting its expression in NSCLC cell lines and tissues. In this study, *in vivo* stimulation of MDSCs increase was achieved through Plxpsp-mGM-CSF transfection. Tumor transplantation experiments involved A549, A549+GM-CSF, and A549+GM-CSF-siAPE1 cell lines. We further used transcriptomics methods to analyze potential targets of APE1 and screened for molecules related to regulating MDSC. Our findings indicate that APE1 knockdown impedes tumor progression and mitigates inflammatory infiltration within lung cancer tissues. Strategies directed at the APE1/MDSC axis present a promising avenue for the prevention and treatment of lung cancer, offering novel insights into the management of NSCLC.

## RESULTS

### Differential expression of APE1 in lung cancer

**Figure 1:**
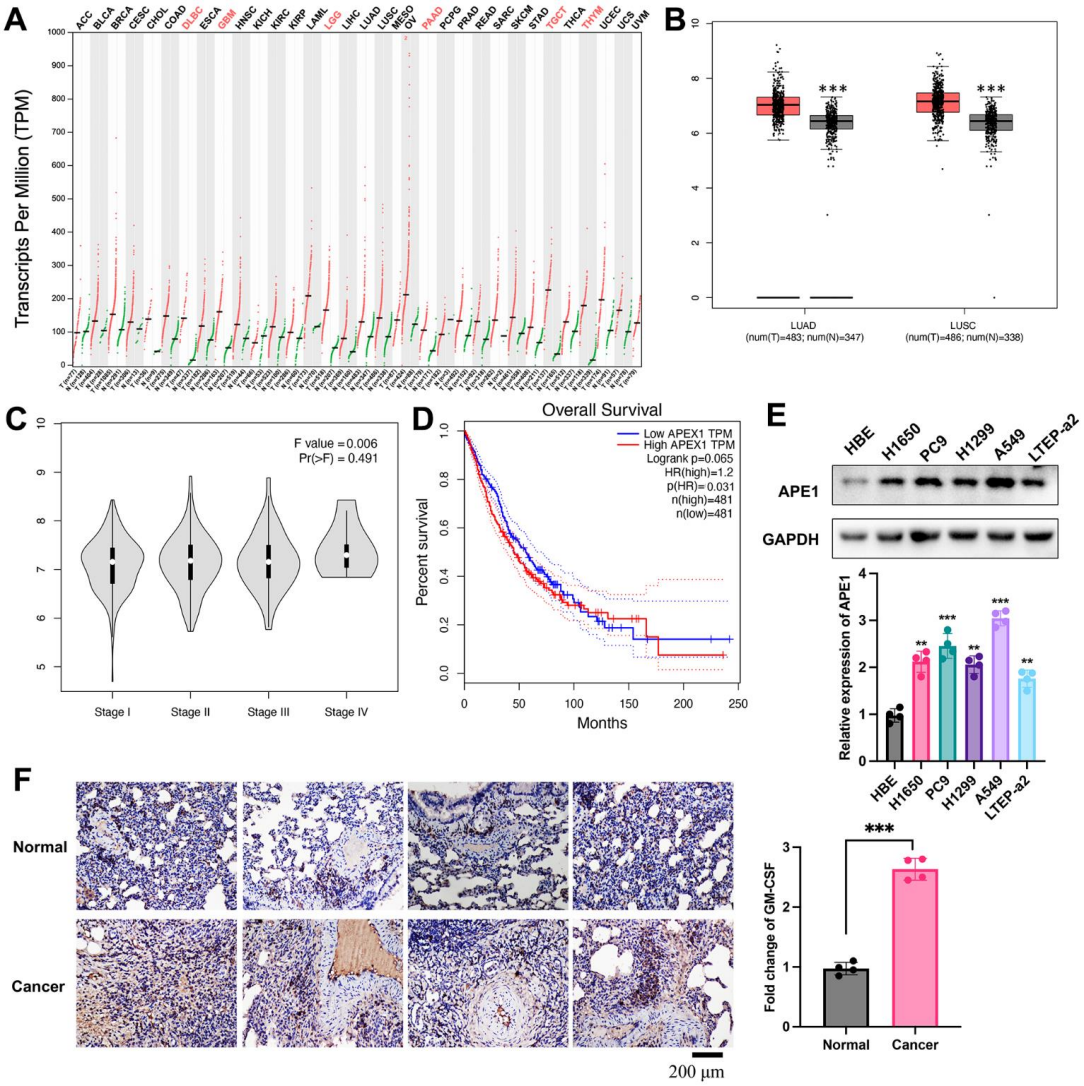
**Differential expression of APE1 in lung cancer.** (**A**, **B**) Analysis of APE1 expression in various tumor types and normal tissues using GEPIA and TCGA databases. (**C**, **D**) Negative correlation of decreased APE1 expression with tumor stage and positive correlation with higher survival rate based on GEPIA analysis. (**E**, **F**) APE1 expression in cancer cells and tissues measured by Western blotting and IHC staining. **p < 0.01, ***p < 0.001.

Utilizing the GEPIA and TCGA databases, we conducted an analysis of APE1 expression across various tumor types, revealing distinct patterns. Notably, lung cancer tissues exhibited significantly lower APE1 expression levels (Figure 1A, 1B). The diminished APE1 expression correlated negatively with tumor stage (Figure 1C) and positively with a higher survival rate (Figure 1D). Furthermore, the normal lung cells (Figure 1E) and tissues (Figure 1F) displayed significantly lower APE1 expression.

### GM-CSF overexpression in A549 and tumor growth suppression by siAPE1

**Figure 2:**
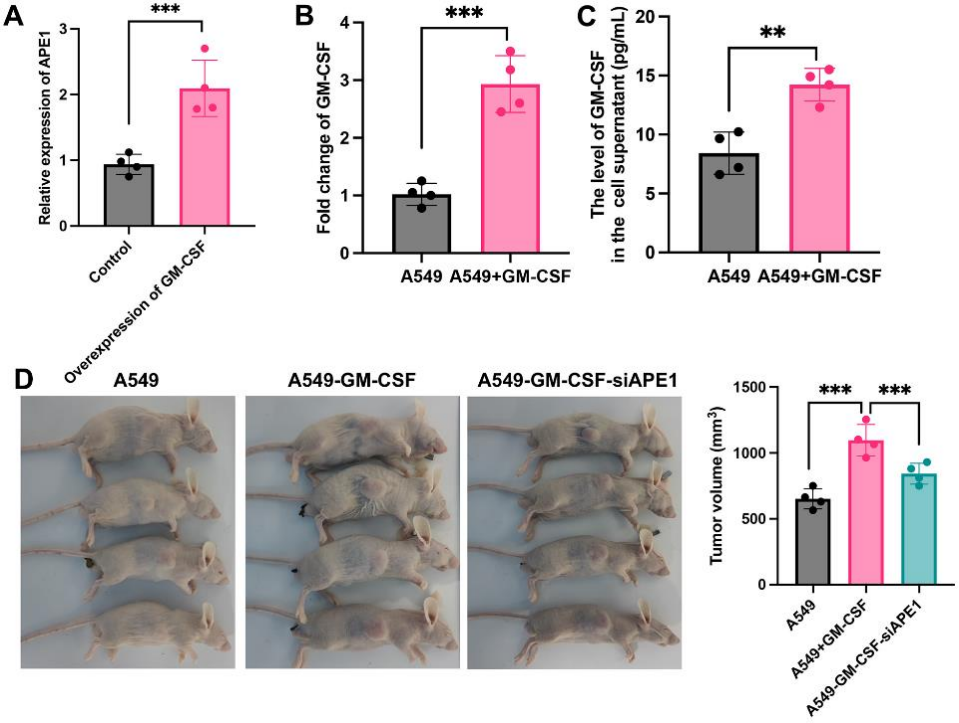
**GM-CSF overexpression in A549 and tumor growth suppression by siAPE1.** (**A**) Establishment of high GM-CSF expression in HBE cells (n=4); (**B**) Significant GM-CSF expression in cells after incubation with supernatant containing high levels of GM-CSF (n=4); (**C**) High GM-CSF expression in supernatant after incubation with supernatant containing high levels of GM-CSF (n=4); (**D**) Tumor growth inhibition by siAPE1 transfection compared with A549+GM-CSF group. **p < 0.01, ***p < 0.001.

A549 cells were transfected with Plxpsp-mGM-CSF for 48 hours, resulting in a substantial increase in GM-CSF expression in the supernatant (Figure 2A). The supernatant, rich in GM-CSF, was employed to incubate A549 cells, leading to notable elevation in both intracellular and extracellular GM-CSF levels (Figure 2B, 2C). Consequently, A549 cells with heightened GM-CSF expression were successfully established. Notably, siAPE1 transfection significantly inhibited tumor growth compared with A549+GM-CSF group (Figure 2D).

### Knockdown of APE1 regulated the immune system of lung tumor mice

**Figure 3:**
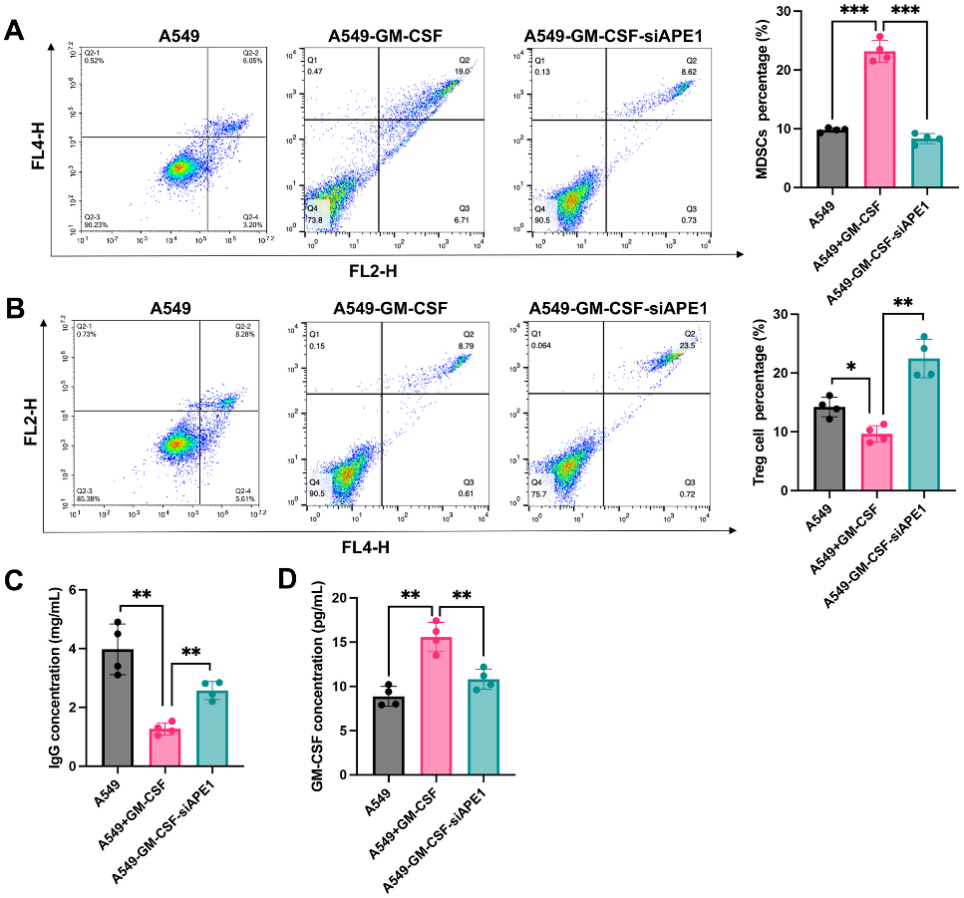
**Knockdown of APE1 regulated the immune system of lung tumor mice.** (**A**) Significant inhibition of MDSCs in the A549+GM-CSF-siAPE1 group compared to A549+GM-CSF; (**B**) Analysis of Treg cells by flow cytometry; (**C**) Analysis of IgG levels by ELISA; (**D**) Analysis of GM-CSF levels. *p < 0.05, **p < 0.01, ***p < 0.001.

**Figure 4:**
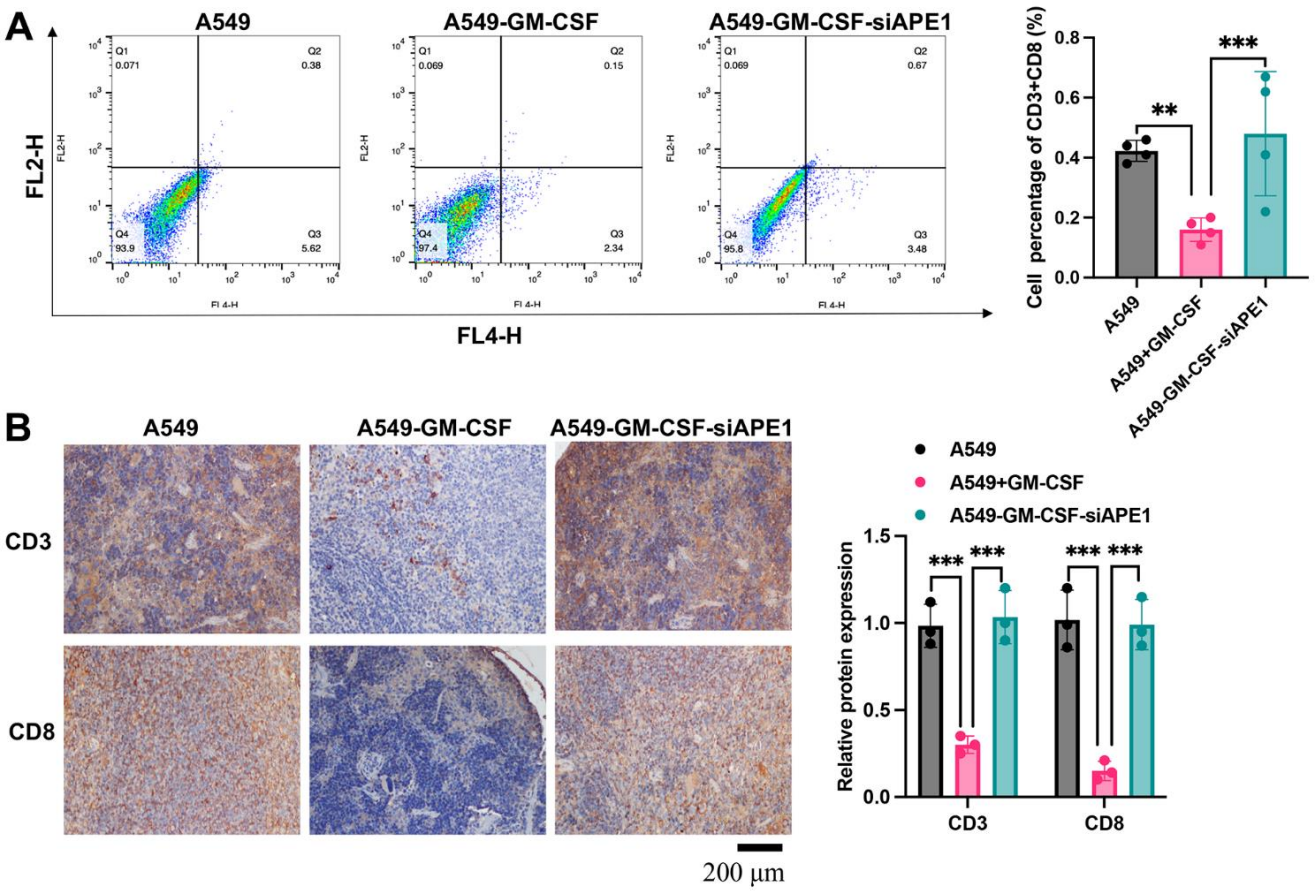
**APE1 knockdown enhances CD3+ and CD8+ levels.** (**A**) Analysis of CD3+ and CD8+ cells by flow cytometry; (**B**) Analysis of CD3+ and CD8+ cells by IHC staining. **p < 0.01, ***p < 0.001.

The A549+GM-CSF group exhibited a significant increase in MDSC levels compared to the A549 group (Figure 3A). However, MDSCs were markedly suppressed in the A549+GM-CSF-siAPE1 group. Furthermore, lower levels of Treg cells and IgG were observed in the A549+GM-CSF group, but these were significantly enhanced in the A549+GM-CSF-siAPE1 group (Figure 3B, 3C). Additionally, GM-CSF levels increased in the A549+GM-CSF group but were attenuated after siAPE1 treatment (Figure 3D). Reduced levels of CD3 and CD8 in peripheral blood and spleen tissues were observed in the A549+GM-CSF group, but siAPE1 transfection markedly increased CD3 and CD8 levels (Figure 4A, 4B). These results suggest that A549+GM-CSF transplantation leads to increased tumor growth and immune system suppression, while siAPE1 transfection inhibits tumor development and strengthens the immune system.

### APE1 knockdown enhances anti-tumor immunity in subcutaneous A549 mouse model

**Figure 5:**
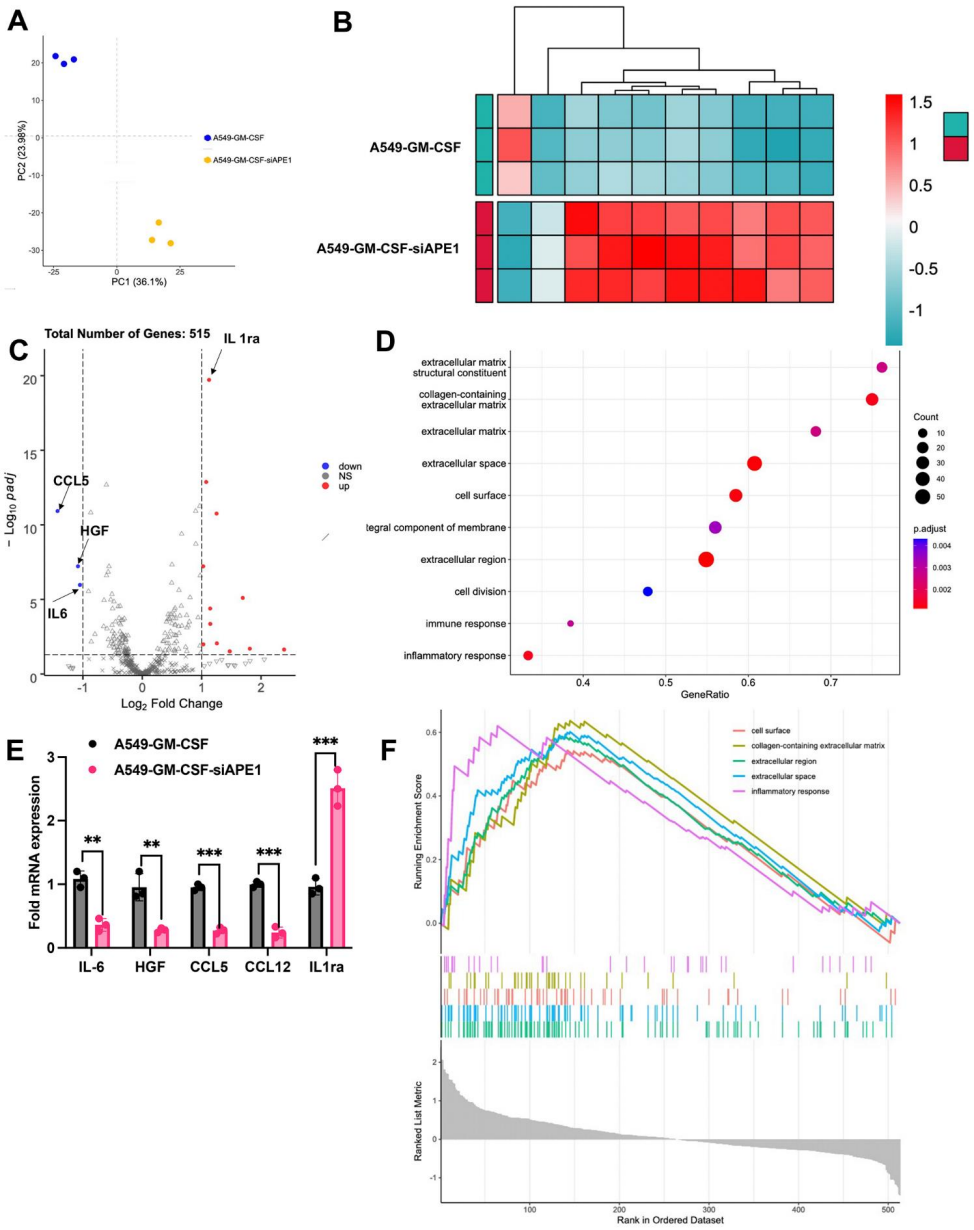
**APE1 knockdown enhances anti-tumor immunity in subcutaneous A549 mouse model.** (**A**) PCA analysis; (**B**, **C**) Analysis of differential expression genes; (**D**) KEGG pathway analysis; (**E**) Validation of MDSC-related cytokine expression by RT-PCR; (**F**) Summary of transcriptome analysis. **p < 0.01, ***p < 0.001.

Principal Component Analysis (PCA) demonstrated clear clustering of samples within each group, distinctively separating the A549+GM-CSF and A549+GM-CSF-siAPE1 groups (Figure 5A). Comparative gene expression profiling revealed significant differences, with enriched immune response and inflammatory response pathways observed in the A549+GM-CSF-siAPE1 group (Figure 5B–5D). Notably, siAPE1 treatment resulted in the downregulation of critical genes associated with MDSC accumulation, such as HGF and CCL5 (Figure 5C). RT-qPCR analysis of isolated lung tumor tissues confirmed lower levels of Pro-MDSC cytokines (HGF, CCL5, IL-6, CCL12) in the A549+GM-CSF-siAPE1 group, with a concomitant upregulation of the anti-MDSC cytokine IL-1ra (Figure 5E) in the group A549+GM-CSF-siAPE1, compared with group A549+GM-CSF.

### APE1 knockdown inhibits cell viability in A549 and H1650 cells

**Figure 6:**
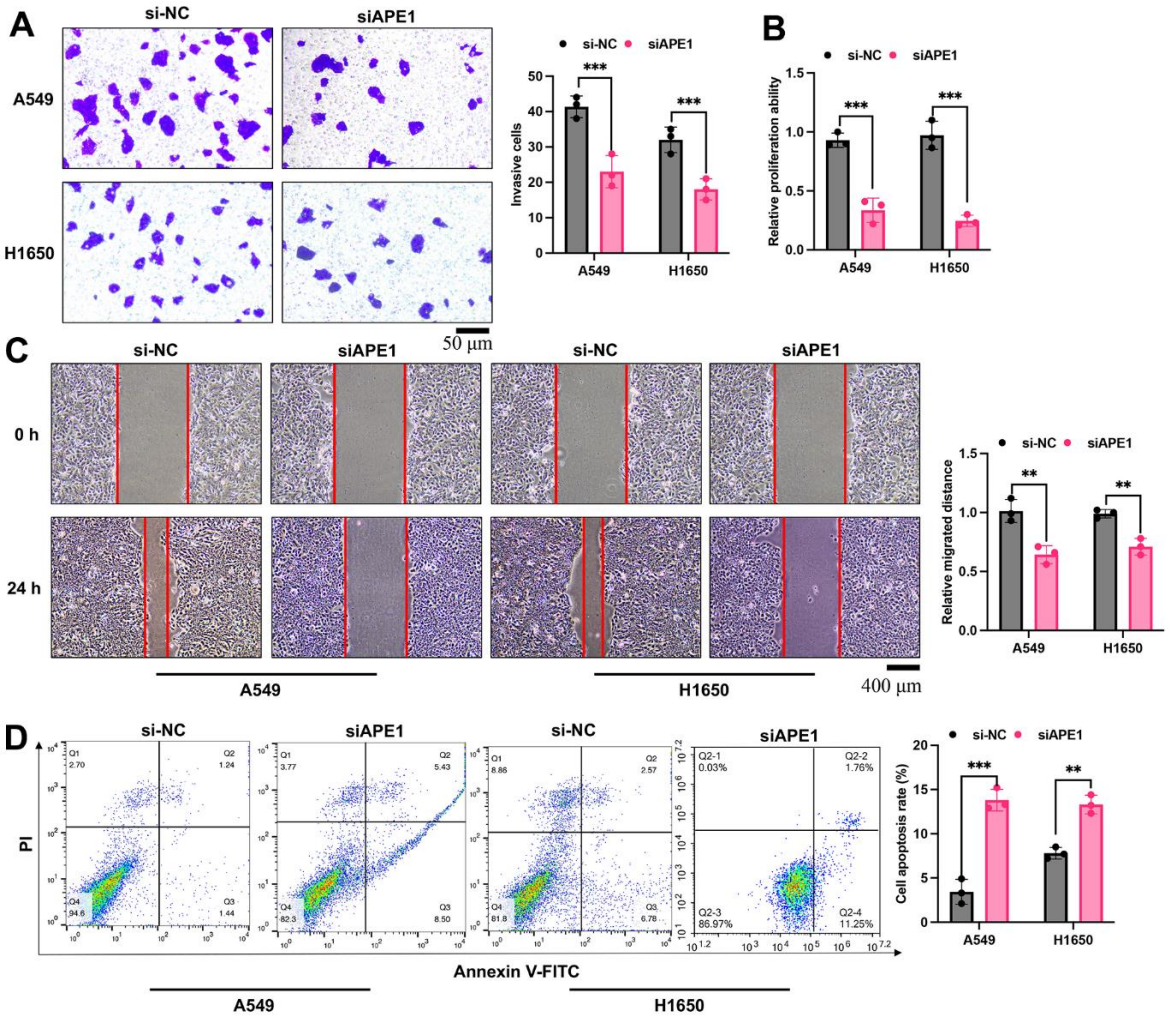
**APE1 knockdown inhibits cell viability in A549 and H1650 cells.** (**A**–**D**) Detection of cell invasion, proliferation, migration, and apoptosis by Transwell, CCK8, wound healing, and flow cytometry assays. **p < 0.01, ***p < 0.001.

siAPE1 significantly suppressed cell invasion, proliferation, and migration in both A549 and H1650 cells compared to the si-NC treated group (Figure 6A–6C). Moreover, APE1 knockdown markedly increased cell apoptosis relative to the si-NC treated group (Figure 6D).

## DISCUSSION

Lung cancer is the most common and deadliest malignancy worldwide. The majority of NSCLC patients present late-stage disease, widespread tumor involvement, and/or metastasis, rendering them ineligible for surgical intervention [2, 15]. Consequently, systemic chemotherapy plays a pivotal role in lung cancer treatment. The occurrence of resistance limits the clinical benefits of TKIs [16]. The optimal treatment strategy after progression, the biological characteristics of EGFR-mutated NSCLC, and the specific mechanisms underlying resistance to targeted therapies remain unclear.

The mechanism by which MDSCs promote immune escape in tumors is complex. MDSCs can upregulate the expression of PD-L1 on their surface, binding to PD-1 on the surface of T cells, or induce T cell apoptosis by expressing galectin-9, which binds to the immune globulin and mucin domain protein 3 on T cells [17]. In the inflammatory and hypoxic microenvironment of tumor-infiltrated tissues, MDSCs can secrete transforming growth factor-beta, upregulate nitric oxide, produce reactive oxygen species, and enhance the activity of arginase-1, among other mechanisms. These actions promote the differentiation of regulatory T cells [18]. In this research, overexpression of GM-CSF could stimulate the high expression of MDSCs. we found that the increased MDSCs in the group A549+GM-CSF was greatly inhibited by siAPE1 (Figure 3A). In addition, knockdown of APE1 orchestrated a modulation of the immune system in lung tumor mice, characterized by a reduction in MDSCs and an augmentation of Treg cells, IgG, CD3, and CD8 (Figures 3, 4).

MDSCs-produced chemokines, such as CCL12 and CCL5, can also induce Tregs to infiltrate into tumors, exerting their immunosuppressive effects [19]. MDSCs may also influence the immune function of natural killer (NK) cells, macrophages, dendritic cells (DCs), and B cells through various mechanisms, thus promoting immune tolerance and leading to tumor immune escape [20]. It is evident that MDSCs affect multiple aspects of anti-tumor immunity, severely impeding the body’s immune response to cancer [21]. In this research, the differential genes between group A549+GM-CSF and A549+GM-CSF-siAPE1 were screened and validated (Figure 5B–5E). The suppression of MDSCs-produced chemokines (HGF, CCL5, IL-6, CCL12) by siAPE1 was proved.

APE1 is regulated at the epigenetic, transcriptional, and post-transcriptional levels, and it can itself regulate the expression of multiple genes [22]. As a multifunctional protein, dysregulation of APE1 is associated with tumor occurrence, development, tumor angiogenesis, progression, and metastasis [23]. In relation to clinical pathological features, overexpression of APE1/Ref-1 is correlated with chemotherapy resistance, poor prognosis, and shorter survival periods [24, 25]. This study revealed that patients with low levels of APE1 expression exhibit better overall survival, suggesting that high APE1 expression can serve as an adverse prognostic factor for tumor. Our findings in this research are in line with previous study. Therefore, APE1 is considered a highly promising therapeutic target for cancer treatment. The regulation of NSCLC by APE1 has been reported. APE1 controls DICER1 expression in NSCLC through miR-33a and miR-130b [26]. Small-molecule inhibition of APE1 induced apoptosis, pyroptosis, and necroptosis in NSCLC [27]. However, we firstly demonstrated that APE1 could regulate NSCLC via affecting MDSCs-induced immune disorders.

## Conclusion

Transplantation of A549-GM-CSF resulted in an increase in MDSC levels, contributing to accelerated tumor growth. Conversely, the reduction of MDSCs through APE1 knockdown effectively restrained tumor progression and mitigated inflammatory infiltration in lung cancer tissues. Strategies targeting the APE1/MDSC axis present a novel avenue for the prevention and treatment of lung cancer.

## MATERIALS AND METHODS

### Cell culture

Human bronchial epithelial cells (HBE), PC9, H1299, LTEP-a2, A549, and H1650 cells procured from the American Type Culture Collection (ATCC, Manassas, VA, USA) were employed in the present study. These cells were cultured in Dulbecco’s Modified Eagle Medium (DMEM, Gibco, #12491015, Langley, OK, USA) supplemented with 5% fetal bovine serum (FBS, #26010066, Gibco, USA), 60 μg/ml streptomycin (#ST487, Beyotime, China), and 60 IU penicillin (#V900929, Sigma, USA) at 5% CO_2_ and 37° C.

### Cell transfection

HBE cells underwent transfection with Plxpsp-mGM-CSF (72 h), harboring the granulocyte-macrophage colony-stimulating factor (GM-CSF) gene and conferring resistance to Hygromycin B, through the utilization of lipofectamine 2000 (#11668019, Invitrogen, USA). The resulting supernatant from the transfected HBE cells, containing Plxpsp-mGM-CSF, was subsequently applied to A549 cells for a 24-hour incubation, leading to the designation of these cells as A549+GM-CSF.

### Mouse lung cancer model establishment

A549 (1×10^7^, 0.2 ml), A549+GM-CSF (1×10^7^, 0.2 ml), or A549+GM-CSF-siAPE1 (1×10^7^, 0.2 ml), cells were subcutaneously inoculated into the dorsal region of severe combined immunodeficient (SCID) mice (SPF grade, 4-6 weeks old, 18-25g). After 3 weeks, the mice were euthanized, and tumor volume were analyzed via measuring length, width, and height. All animal procedures were ethically approved by the institutional ethics committee of Fujian Medical University, Fujian Provincial Hospital.

### Immunohistochemical staining (IHC)

Following sacrifice, the spleen was collected, dehydrated samples were paraffin-embedded, and 8 μm sections were prepared after pre-cooling. Antigen retrieval was achieved through microwave heating for 3 minutes, followed by a 2-minute incubation with 3% hydrogen peroxide. Sections were then washed with PBS, blocked with 5% non-fat milk (#MB4219-3, Meilunbio, China), and incubated with primary antibodies. Subsequent steps involved washing, exposure to secondary antibodies for 3 hours, application of DAB chromogenic solution, dehydration, and mounting. Observations were made using an Olympus BX41 microscope (Tokyo, Japan).

### Flow cytometry

Blood samples (200 μL) were collected through eyeball extraction in the third week, with CD3 and CD8 monoclonal antibodies labeled with FITC added for flow cytometry analysis.

### Measurement of IgG and GM-CSF

Peripheral blood samples were obtained at 3 weeks post-tumor implantation and subjected to quantification of IgG (#ab151276, Abcam, UK) and GM-CSF (#BMS612, Invitrogen, USA) levels utilizing the enzyme-linked immunosorbent assay (ELISA) method.

### Measurement of MDSCs and Treg cells

Three weeks after tumor implantation, blood samples were collected by enucleation. Peripheral blood mononuclear cell (PBMC) suspension was prepared using the Percoll method. CD3+ lymphocytes were isolated using anti-CD3 magnetic beads, and CD8+ T lymphocytes were selected via flow cytometry. The remaining liquid underwent separation through magnetic beads and flow cytometry to isolate CD11b+ myeloid-derived suppressor cells (MDSCs). The BD CELL Quest software facilitated subsequent analysis.

### CCK8 assay

Cells in the logarithmic growth phase underwent PBS washing, trypsin digestion, suspension in serum-free medium, and adjustment to a cell concentration of 1×10^5^/ml. These cells were then plated into a 96-well plate and cultured for 24 hours. Following incubation with the CCK8 reagent (Beyotime, #C0038, China) for 30 minutes, the cell proliferation ability was quantified.

### Transwell assay

A total of 3×10^5^ cells were seeded in the upper chamber (Costar, Dallas, TX, USA) with a matrix gel (1:5 diluted, Corning, Inc. Corning, NY, USA) and FBS-free medium. Cells were seeded into the upper chamber of the Transwell (2 × 10^4^ cells/insert), and DMEM 10% FBS was added to the lower chamber. After 48 hours, invasive cells in the lower chamber were fixed with 4% methanol and stained with crystal violet. Cells that invaded through the pores to the lower surface of the filter were counted under a microscope. Three invasion chambers were used per condition, and the total number of cells from the three filters was averaged.

### Wound healing assay

For the wound healing assay, cells were trypsin-digested, centrifuged, and then inoculated into a 6-well plate at a density of 106 cells per well. Following 24 hours of culture in a cell incubator to attain 90% confluence, a 200 μL pipette was employed to create a scratch on the cell monolayer. After rinsing twice with PBS to eliminate non-adherent cells, the gap left at the scratch became clearly visible. Fresh serum-free culture medium was added, and the distance between the wounds was measured at 0 and 24 hours, with subsequent analysis of migration distance.

### Bioinformatics analysis

Gene expression and survival rate analyses were conducted using GEPIA (http://gepia.cancer-pku.cn/) and TCGA (https://www.cancer.gov/about-nci/organization/ccg/research/structural-genomics/tcga). RNA purity and integrity assessments were performed using a NanoPhotometer spectrophotometer and an Agilent 2100 bioanalyzer, respectively. The NEBNext® Ultra™ RNA Library Prep Kit for Illumina was used for library construction. Sequencing by Synthesis was employed for sequencing, and StringTie (version 1.3.3b) facilitated new gene prediction. Differential expression analysis between two comparison groups was carried out using DESeq2 software, with adjusted P-values and |log2foldchange| serving as the threshold for significance.

### Western blotting

Western blot analysis was conducted by lysing proteins with RIPA lysis buffer supplemented with a protein phosphatase inhibitor. Protein concentration was determined using the BCA method (#A045-4-1, Nanjing Jiancheng Bioengineering Institute, Nanjing, China), and equivalent amounts of protein for each experimental group were loaded onto 10% SDS-PAGE gels. Subsequently, proteins were transferred onto a PVDF membrane. The membrane was blocked with TBST containing 5% non-fat milk for 2 hours. Following the blocking step, primary antibodies (#ab189474, #ab9485) were incubated overnight at 4° C, and secondary antibodies were applied for 2 hours at room temperature. Detection of target genes was accomplished using an enhanced chemiluminescence detection kit from Thermo Fisher Scientific, USA. ImageJ software was utilized for the analysis of protein bands.

### RT-PCR

For RT-PCR analysis, RNA extraction from tissues was conducted using Trizol (#R0016, Beyotime). The Nanodrop 2000 spectrophotometer (Thermo Fisher Scientific, USA) was employed to assess RNA purity. Reverse transcription was carried out using the Takara PrimeScript RT reagent kit with gDNA eraser kit (#RR047A). Subsequent RT-PCR was executed utilizing the Bio-Rad CXF96 system. The relative expression levels of the target gene were determined through the 2^-ΔΔCT^ method. The program was set as follows: denaturation at 93° C for 3 min, cycling amplification stage: 93° C for 40 s, 58° C for 30 s, 72° C for 60 s (cycling 35 times), holding at 72° C for 7 min. The primers used in this research were listed below: IL-6 (F- AGACAGCCACTCACCTCTTCAG; R-TTCTGCCAGTGCCTCTTTGCTG); HGF (F-GAATGCATGACCTGCAACGG; R-TGTCGGGATATCTTTCCGGC); CCL5 (F- CCTGCTGCTTTGCCTACATTGC; R-ACACACTTGGCGGTTCTTTCGG); CCL12 (F-GCTACAGGAGAATCACAAGCAGC; R- ACGTCTTATCCAAGTGGTTTATGG).

### Statistical analysis

Data were presented as mean ± SD. Statistical analyses were executed using SPSS software version 18. A p-value < 0.05 was deemed statistically significant, and T-tests were used for analysis between 2 groups, and ANOVA was employed for statistical analysis among groups more than 2. p-values were adjusted for multiple comparisons to avoid false discovery reporting in this research.

### Availability of data and material

The data and material used to support the findings of this study are included within the manuscript and Supplementary Files.
